# Implication of mitochondria-derived ROS and cardiolipin peroxidation in N-(4-hydroxyphenyl)retinamide-induced apoptosis

**DOI:** 10.1038/sj.bjc.6600356

**Published:** 2002-06-17

**Authors:** A Asumendi, M C Morales, A Alvarez, J Aréchaga, G Pérez-Yarza

**Affiliations:** Department of Cell Biology and Histology, School of Medicine and Dentistry, University of The Basque Country, Leioa- 48940, Bizkaia, Spain

**Keywords:** N-(4-hydroxyphenyl)retinamide, apoptosis, reactive oxygen species, mitochondria, cardiolipin peroxidation, cytochrome *c*

## Abstract

We have studied the effect of N-(4-hydroxyphenyl)retinamide on either malignant human leukaemia cells or normal cells and investigated its mechanism of action. We demonstrate that 4HPR induces reactive oxygen species increase on mitochondria at a target between mitochondrial respiratory chain complex I and II. Such oxidative stress causes cardiolipin peroxidation which in turn allows cytochrome *c* release to cytosol, caspase-3 activation and therefore apoptotic consumption. Moreover, this apoptotic pathway seems to be bcl-2/bax independent and count only on malignant cells but not normal nor activated lymphocytes.

*British Journal of Cancer* (2002) **86**, 1951–1956. doi:10.1038/sj.bjc.6600356 www.bjcancer.com

© 2002 Cancer Research UK

## 

Recently, retinoids have generated interest as potential therapeutic and preventive agents for the treatment of cancer. Among the synthetic retinoids with minimal clinical toxicity, N-(4-hydroxyphenyl)retinamide (4HPR), also named fenretinide, has emerged as one of the most promising alternatives to the natural–more toxic–retinoids. 4HPR inhibits carcinogenesis and has therapeutic effects in a variety of animal cancer models. Trials in humans are also presently under way with 4HPR (Ulukaya and Wood, 1999). These anticancer activities seem to be related with their ability *in vitro* to inhibit tumour cell growth and to induce apoptosis (Delia *et al*, 1993; Kalemkerian *et al*, 1995). Recently, reactive oxygen species (ROS) have been implicated in the mediation of apoptosis by 4HPR in human cancer cells (Delia *et al*, 1997; Maurer *et al*, 1999; Suzuki *et al*, 1999; Hail and Lotan, 2001), but at least one mechanism independent to ROS, which involved RAR-mediated pathway, has also been reported to be involved (Sun *et al*, 1999).

Accumulating evidence points to a central role of mitochondria during apoptosis. Alterations in mitochondrial physiology, such as dissipation of the inner transmembrane potential and/or release of intermembrane proteins through the outer membrane, have been described in many apoptotic responses (Loeffler and Kroemer, 2000). Among the released proteins, caspases (mainly 2, 3 and 9), caspase activators (cytochrome *c*, hsp10) as well as a caspase-independent death effector AIF (apoptosis inducing factor) have been identified, and they are ultimately responsible for apoptotic cell consumption (Loeffler and Kroemer, 2000).

In this study, we contribute with new data to the mechanisms by which 4HPR induces cell death in cancer cells, involving cardiolipin peroxidation as the cause of cytochrome *c* release and caspase-3 activation.

## MATERIALS AND METHODS

### Cell cultures and isolation of peripheral lymphocytes

CCRF–CEM human acute lymphoblastoid leukaemia cells were routinely grown in RPMI 1640 supplemented with 10% heat inactivated foetal calf serum (FCS), 100 μg ml^−1^ gentamicine and 2 mM L-glutamine. Human peripheral blood mononuclear cells from normal volunteers were obtained after Ficoll-Paque (Pharmacia LKB, Uppsala, Sweden) density gradient centrifugation and differential adhesion. Cells were cultured in 10% FCS and RPMI 1640 containing L-glutamine, 50 μM 2-Mercaptoethanol. To obtain activated lymphocytes, 20 μg ml^−1^ phytohemagglutinin-M (Roche Molecular Biochemicals) were added to culture, proliferating cells were collected after 48 h, washed and seeded at 1×10^6^ cells ml.

### Cell survival assay

A standard XTT assay (Roche Molecular Biochemicals, IN, USA) was used to determine cell survival. Cells were plated in 96-well plates at a density of 1×10^6^ cells per ml (four wells per experimental condition), exposed to a range of concentrations of 4HPR and processed according to the manufacturer's instructions. In some experiments, 10 mM N-acetyl-cysteine (NAC) or 100 μM vitamin E (Sigma) were added 2 h before 4HPR addition. Absorbance at 480 nm was determined for each well, and cell survival percentages were calculated in each experiment in relation to controls–non treated–cells.

### Flow cytometry

The Annexin V-FITC apoptosis detection kit (Calbiochem, Oncogene) was used to distinguish between apoptosis and necrosis. Cells were labelled after treatments according to the manufacturer's instructions and analysed by flow cytometry. Viable cells do not bind Annexin V-FITC or propidium iodide. Early apoptotic cells bind Annexin V-FITC but exclude propidium iodide. Necrotic and apoptotic cells in terminal stages (secondary necrosis) are both Annexin V-FITC and propidium iodide positive. Reduction of mitochondrial membrane potential was evaluated by Rhodamine 123 labelling (Molecular Probes, Eugene, OR, USA). After drug exposure cells were incubated with 5 μg ml^−1^ Rh 123 in RPMI 1640 containing 10% FCS for 30 min at 37°C. Then cells were washed twice with PBS and incubated for 30 min with 10% FCS RPMI 1640. After washing, 5 μg ml^−1^ IP were added and viable cells were analysed by flow cytometry. At least 10 000 viable cells per sample were individually analysed for quantitative fluorescence using a Coulter EPICS ELITE ESP flow cytometer (EPICS division Coulter Corp.).

### Western blotting

Pellets were resuspended in Laemmli's SDS–PAGE sample buffer to a 1× and the viscosity of the lysates was reduced by shearing the DNA three times through a 26 needle. After boiling for 5 min, 30 μg of each protein samples were subjected to electrophoresis in 15% polyacrylamide gels in the presence of 0.1% sodium dodecyl sulphate and transferred to nitrocellulose membranes. The nonspecific binding sites on the membrane were first blocked with 5% (w v^−1^) dried milk in 20 mM Tris-HCl (pH 8.0) containing 0.1% (v v^−1^) Tween 20 (TTBS) for 1 h at room temperature. After three washes with TTBS, the membrane was incubated with anti-caspase-3, anti-bcl-2 (Santa Cruz Biotechnology, Inc., CA, USA) or anti-bax (R&D Systems Minneapolis, MN, USA) antibodies diluted 1:100 in 2.5% (w v^−1^) dried milk in TTBS for 90 min at room temperature. The membranes were then washed three times in TTBS, and incubated with anti-mouse or anti-rabbit IgG-peroxidase conjugate diluted 1 : 3000 in 1% (w v^−1^) dried milk in TTBS for 1 h at room temperature. The membrane was then washed four times with TTBS. The binding of the antibodies were probed by the chemiluminescence ECL method according to the manufacturer's instructions (Amersham Life Science Inc., Arlington Heights, IL, USA). For the detection of cytochrome *c* release from mitochondria, cytosolic extracts were used. After treatments, cells were centrifugated, resuspended in buffer I (220 mM mannitol, 68 mM sucrose, 50 mM PIPES-KOH pH 7.4, 50 mM KCl, 5 mM EGTA, 2 mM MgCl_2_, 1 mM DTT) and placed on ice during 10 min. Cells were centrifugated at 1000 **g** for 5 min and then 30 μl of buffer I supplemented with leupeptin 1 μg ml^−1^, aprotinin 1 μg ml^−1^, and pepstatin 1 μg ml^−1^ were used to suspend the cells. The cells were then disrupted by homogenisation on ice in a teflon dounce (100 strokes), the homogenate was subjected to centrifugation at 16 000 **g** for 1 min, and then the supernatant was collected and subjected to another centrifugation at 16 000 **g** for 30 min to obtain a cytosolic extract. Samples of the cytosolic extracts containing 40 μg protein were subjected to electrophoresis using 15% polyacrylamide gel, transferred and developed as described. Anti-cytochrome *c* monoclonal antibody was purchased by BD Pharmingen (San Diego, CA, USA) and used at 1 : 500 dilution.

### Measurement of intracellular generation of ROS

**Figure 6:**
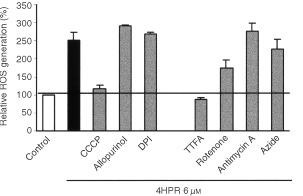
Effects of various inhibitors on the 4HPR-induced ROS generation in CCRF–CEM cells. Cells were treated with 4HPR and/or inhibitors for 30 min and then ROS generation was measured. Relative percentages with respect to controls (basal production, white bar) are shown. The black bar represents ROS production of 4HPR treated cells. The mean±s.d. of three independent experiments is shown.

We used the oxidation-sensitive fluorescent dye 2′, 7′-dichlorodihydrofluorescein diacetate (DCFH-DA, Molecular Probes, Eugene, OR, USA) to measure the production of reactive oxygen species, mainly hydrogen peroxide and hydroxyl radicals. Cells seeded in 96-well culture plates were exposed to 4HPR alone or in combination with antioxidants. Vitamin E (100 μm) or NAC (10 mM) were added 2 h before the drug. In some cultures (see Figure 6

) the following inhibitors were added in combination with 4HPR: the uncoupler, carbonylcyanide m-chlorophenylhydrazone (CCCP) 10 μM; NADPH inhibitor DPI 2 mM; xanthine/xanthine oxidase inhibitor, allopurinol 1 mM; MRC complex II inhibitor, thenoyltrifluoroacetone (TTFA) 1 mM; MRC complex III inhibitor, antimycin A 2 μg ml^−1^ (all were purchased from Sigma Chemical Co). After 30 min cells were washed and incubated at 37°C with 10 μg ml^−1^ DCFC-DA for 20 min. After washing twice, fluorescence intensity was measured at 530 nm after excitation at 485 nm in a FL500 fluorimeter (Bio-Tek Instruments). Four wells were used for each treatment.

### Measurement of cardiolipin peroxidation

10-N-nonyl-Acridin Orange (NAO, Molecular Probes, Inc.), which binds to mitochondria-specific cardiolipin, was used for this purpose. Decreases in the fluorescence of NAO in apoptotic cells have been reported to reflect the peroxidation of intracellular cardiolipin (Nomura *et al*, 2000) because the fluorochrome loses its affinity for peroxidised cardiolipin. Cells were seeded at a final concentration of 1×10^6^ cells ml^−1^ in 96-well tissue culture plates, treated with different concentrations of 4HPR, washed and labelled with 10 μg ml^−1^ NAO for 20 min. In some cultures 100 μM vitamin E was added 2 h before 4HPR addition. After washing twice, fluorescence emited by cardiolipin-bounded NAO was measured at 530 nm–excitation at 485 nm–in a FL500 fluorimeter (Bio-Tek Instruments). Four wells were used for each experimental condition.

### Statistics

The results were expressed as the mean±s.d of at least three independent experiments. Student's two-tailed, unpaired *t*-test was used, and values of *P* <0.05 were considered to be significant.

## RESULTS

**Figure 1:**
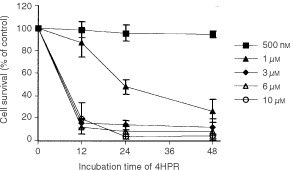
Dose- and time-dependent effect of 4HPR on cell survival of human CCRF–CEM cells. The mean±s.d of three independent experiments are shown.

**Figure 2:**
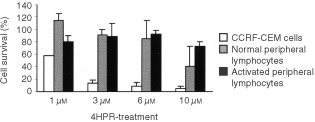
Effect of 4HPR on of peripheral lymphocytes and malignant CCRF–CEM cells survival. Percentages of cells treated for 24 h with increasing doses of 4HPR are shown. The mean±s.d of three independent experiments are shown.

A dose–time study was carried out to establish the effect of 4HPR in the survival rate of CCRF–CEM cells (Figure 1

). The lower concentration in which maximum cell death was achieved was 3 μM, a concentration known to be attainable *in vivo* (Formelli *et al*, 1993). At this dose, nearly 90% of the cells were eliminated in 24 h. However, neither normal nor activated peripheral lymphocytes were affected under these conditions (Figure 2

). Only the maximum concentration tested–10 μM 4HPR–had some effect on the survival of normal lymphocytes, being the latter much more resistant to the drug than malignant cells.

**Figure 3:**
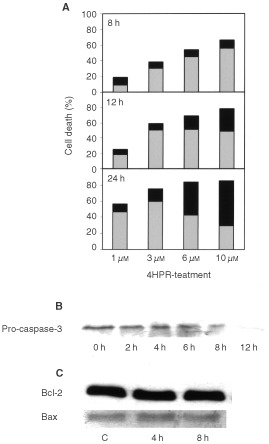
(**A**) Apoptosis and necrosis rates in 4HPR-treated CCRF–CEM cells. Cells were treated with increasing doses of 4HPR for 8, 12 and 24 h and then labelled with Annexin-V and Propidium Iodide as described in Materials and Methods. Apoptosis percentages are represented in grey, and in black percentages of necrotic cells in the same cultures. (**B**) Activation of caspase-3 in 4HPR-treated human CCRF–CEM cells. Blot shows the progressive pro-caspase-3 cleavage along time in 10 μM 4HPR-treated cells. (**C**) Bcl-2 and Bax protein expression in control and 4HPR-treated CEM cells.

4HPR-induced cell death in lymphoblastoid leukaemia cells is caused by apoptosis at pharmacological concentrations (Figure 3A

), but proceeds predominantly by necrosis at higher drug concentrations (10 μM). Ultrastuctural images have confirmed that the enhancement of necrosis percentages is mainly due to secondary necrosis (apoptotic cells in terminal stages) rather than real necrosis. These results are in concordance with the observation that caspase 3 is activated in cells treated with 4HPR (Figure 3B). The blot revealed a progressive cleavage of pro-caspase 3 in the course of time. However, no changes in the expression of apoptosis-related proteins Bcl-2 and Bax have been observed (Figure 3C).

**Figure 4:**
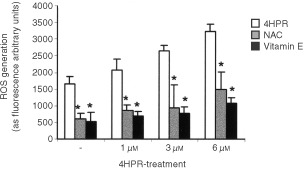
Effect of antioxidants on the generation of reactive oxygen species in 4HPR-treated CCRF–CEM cells. A dose-dependent study. Cells were treated with or without increasing doses of 4HPR for 30 min and then ROS production was measured. In some cultures, the antioxidants NAC and vitamin E were added 2 h before initiating the treatments. The mean±s.d. of three experiments are shown. Differences in ROS production between antioxidant-treated cells and non-treated cells were statistically significant (*P*<0.01).

**Figure 5:**
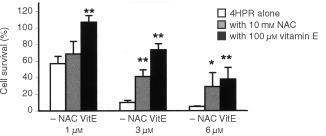
Effect of antioxidants on the survival of 4HPR-treated CEM cells. Cells were treated with 4HPR alone, or in combination with 10 mM NAC or 100 μM vitamin E. Relative percentages with respect to non-treated cells are shown. The means±s.d. of three experiments are shown. Differences in cell survival of antioxidant-treated cells were statistically significant with reference to those only treated with 4HPR (*P<0.05; **P<0.01).

Measurements of cellular fluorescence revealed that 4HPR induced an immediate ROS elevation in CCRF–CEM cells. After 30 min of exposure, a dose-dependent increase in the amount of ROS was observed (Figure 4

). The enhancement of ROS was much higher after 45–60 min of exposure when 4.0-fold increase in ROS was observed at higher doses, and was noticeable just after 5 min of treatment (data not shown). This oxidative stress was inhibited with antioxidants such as N-acetyl-cysteine (NAC) and vitamin E, being the latter more effective at neutralising reactive oxygen species (Figure 4). These antioxidants that are effective at suppressing HPR-induced ROS are also effective at reducing 4HPR-induced apoptosis (Figure 5

). Curiously, vitamin E–which is more powerful than NAC scavenging ROS generated by 4HPR–is also more effective at reducing drug induced cell death. In this sense, vitamin E is able to completely inhibit cell death induced by 1 μM of 4HPR, and reverts from 10 to 75% the cell death induced by 3 μM 4HPR. Results indicate that the antioxidants effect on HPR-induced apoptosis is related with their ability to suppress the enhancement of ROS in 4HPR-treated malignant cells. In contrast, our assays failed to prove enhancement of ROS on 4HPR-treated normal and activated peripheral lymphocytes (data not shown).

To analyse the origin of 4HPR-induced reactive oxygen species, the effect of some radical-producing system inhibitors was tested in the presence of 4HPR (Figure 6). Allopurinol–xanthine/xanthine oxidase inhibitor–and DPI–NADPH oxidase inhibitor-, have no effect on 4HPR-induced ROS increase. However, CCCP, which uncouples electron transfer in mitochondria and inhibits the generation of ROS from MRC, suppressed completely 4HPR-induced oxidative stress. Among the inhibitors of MRC complexes tested, only TTFA–an MRC complex II inhibitor–completely suppressed 4HPR-induced ROS elevation. The MRC complex I inhibitor rotenone inhibited 4HPR-induced ROS generation by 52%, but none of the other inhibitors used (antimycin A which inhibits complex III and azide which inhibits complex IV) caused any effect over the ROS production. These results indicate that 4HPR stimulated ROS production upstream of complex III, preferentially between complexes II and III.

To study if peroxidation of cardiolipin (CL) took place in 4HPR-treated cells, binding of NAO–a fluorescent probe that binds specifically to CL–was measured in treated CEM cells (Table 1

**Table 1:**
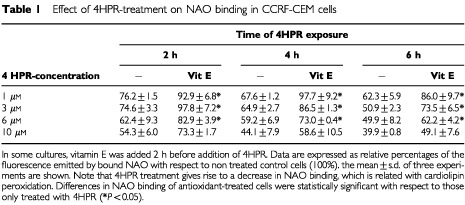
Effect of 4HPR-treatment on NAO binding in CCRF-CEM cells

). Results showed a dose- and time-dependent decrease of NAO binding to 4HPR-treated cells (Table 1), which reflect the peroxidation of mitochondrial CL. Fluorochrome binding reduction was significant after 2 h of 4HPR-treatment with reference to non treated cells, and a decrease of 50–60% in NAO binding was observed at high doses (10 μM). At lower doses (3 μM), about 50% of NAO binding capacity to CL is lost as early as at 6 h of exposure. The antioxidant vitamin E reduces CL peroxidation in all cases, and it is more effective preventing the oxidation induced by lower doses of 4HPR. It is important to note that mitochondrial transmembrane potential reduction–measured by Rhodamine 123 labelling–was not observed until 4 h after exposure to drug (Figure 7

**Figure 7:**
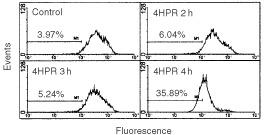
Mitochondrial membrane potential in CCRF-cells treated with 4HPR. Cells treated with 6 μg ml^−1^ 4HPR during short periods of time (1, 3 and 4 h) were labelled with Rhodamine 123 and analysed by flow cytometry. The reduction of transmembrane potential after 4 h of treatment is represented as a shift of the fluorescence peak to lower levels and the percentage of cells in the lower fluorescence category was plotted in the graphs.

). Thus, CL peroxidation precedes mitochondrial potential reduction.

**Figure 8:**
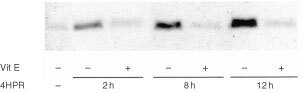
Effect of vitamin E on 4HPR-induced cytochrome *c* release in human CCRF–CEM cells. Cells were incubated without (control) or with 5 μM 4HPR during 2, 8 and 12 h. In some cultures vitamin E was added 2 h before addition of 4HPR.

4HPR induces a release of cytochrome *c* from mitochondria in CEM cells and the amount of cytochrome *c* detected in the cytosol was increasing along the 4HPR-treatment time (0, 2, 8 and 12 h) (Figure 8

). Moreover, this release is clearly reduced in all cases when the vitamin E is added to the cultures. This result shows that 4HPR induces cytochrome *c* release in an antioxidant-sensitive pathway.

## DISCUSSION

Our results indicate that at pharmacological doses, 4HPR causes apoptosis in human acute lymphoblastoid leukaemia cells CCRF–CEM (Figure 3A,B), and that its proapoptotic properties are related with the ability to produce ROS at mitochondrial level. The increase in ROS has been proposed as one mechanism through which 4HPR could exert its proapoptotic effects (Delia *et al*, 1997; Maurer *et al*, 1999; Suzuki *et al*, 1999; Wu *et al*, 2001). The fact that antioxidants NAC and vitamin E decrease both 4HPR-induced ROS production (Figure 4) and cell death (Figure 5), demonstrate that ROS are critical in mediating apoptosis in CEM cells. Also, our studies revealed that 4HPR exerts different effects on either malignant lymphoblasts or normal lymphocytes. 4HPR does not stimulate ROS production on normal or activated peripheral lymphocytes, and subsequently these cells seem to be significantly more resistant to 4HPR than tumour cells (Figure 2). This specificity of retinamide towards malignant cells and not normal cells confers 4HPR an important characteristic as a therapeutic agent. The more or less sensitivity of cells to 4HPR may be due to the status of endogenous antioxidant systems. The major source of ROS in eukariotic cells are the mitochondrial respiratory chain components (Fernández-Checa *et al*, 1998), and the radical-generating enzymes xanthine/xanthine oxidase (McCord, 1985) and NADPH oxidase (Trudel *et al*, 1991). Among all inhibitors of such ROS source used, only those exerting their action on mitochondria have some effect on the 4HPR-induced oxidative stress (Figure 6). Blocking ATP synthesis and ROS generation by MRC with CCCP–an uncoupler of electron transfer–4HPR-induced oxidative stress is completely reduced, which indicates mitochondrial participation. In this sense Hail and Lotan (2001) have demonstrated recently that the pro-oxidant effect of 4HPR is associated with mithochondrial redox metabolism in human cutaneous carcinoma cells. To reinforce the finding that electron flow at MRC is the major site of ROS generation by 4HPR, we inhibited electron flow at complexes I to IV, and the results illustrated that ROS generation source is probably located downstream MRC complexes I and II, and upstream complex III (Figure 6). These results are in accordance with those reported by Suzuki *et al* (1999) in cervical carcinoma cells, which point to a target site of 4HPR action in ROS generation located between complexes II and III. Additional studies are required to determine whether ROS increase at these sites is a general phenomenon or not in 4HPR-induced apoptosis.

Nowadays it is generally accepted that alterations of the mitochondrial function, such as a reduction of the inner transmembrane potential and release of proteins through the outer membrane, constitute an important feature of apoptosis. Our studies show that in 4HPR treated cells, ROS enhancement–perceptible just after 5 min but significant after 30 min–is generated earlier than mitochondrial membrane reduction (observed after 4 h, Figure 7). Therefore, it is likely that mitochondrial alterations observed are due to the direct action of 4HPR-induced ROS. ROS are able to open mitochondrial transition pores leading to an interruption of mitochondrial potential and release of apoptogenic factors (Kroemer *et al*, 1997). However, the mechanism responsible is not defined. Pro- and anti-apoptotic members of the Bcl-2 family can control the mitochondrial transition pores in some systems (Loeffler and Kroemer, 2000). No participation of Bcl-2/Bax in 4HPR-induced cell death was observed in our studies (Figure 3C). Expression of other proteins tested such as Bak and Bcl-xl also remain unaltered in 4HPR-treated CEM cells (data not shown). Lipid peroxidation, which is known to occur during cell death after stimulation with pro-oxidants (Fernández-Checa *et al*, 1998; Nomura *et al*, 2000), could be one of the causes that originate mitochondrial dysfunction. Our results show that 4HPR-induced mitochondrial ROS increase is the cause of cardiolipin peroxidation as early as 2 h after 4HPR treatment (Table 1), before reduction of mitochondrial membrane potential was evident (Figure 7). Cardiolipin–which is located exclusively in the inner mitochondrial membrane–, has a pivotal role as the boundary lipid of various proteins such as NADH:ubiquinone oxidoreductase, cytochrome *c* oxidase and cytochrome *c* (Hoch, 1992). Specifically, cytochrome *c* associates strongly with cardiolipin. A linkage between mitochondrial lipid peroxidation and cytochrome *c* release has been recently established (Nomura *et al*, 2000). Nomura *et al* (2000) have shown that peroxidation of CL in the mitochondria resulted in the dissociation of cytochrome *c* from mitochondrial inner membranes, the initial step in the release of cytochrome *c* to cytosol. Furthermore, cardiolipin is essential for the activity of ANT (adenine nucleotide translocator) (Hoffmann *et al*, 1994), a protein of permeability transition pores, and therefore cardiolipin peroxidation could explain why ROS are able to open the mitochondrial permeability transition pores. Release of cytochrome *c*, which occurs following a variety of death stimuli, has been shown to activate Apaf-1 (apoptotic protease-activating factor 1), which in turn activates caspases-9 and -3 (Li *et al*, 1997; Zou *et al*, 1997) that finally dismantle the cell. It now seems likely that cardiolipin peroxidation caused by 4HPR-induced ROS (Table 1) triggers cytochrome *c* release (Figure 7) and, in consequence, the observed caspase-3 activation (Figure 3). Reinforcing this idea is the finding that vitamin E–whose major function is to protect the polyunsaturated membrane lipids against free radical attack–appears to be more effective against 4HPR-induced apoptosis than other antioxidants tested (Figure 5). Furthermore, we have demonstrated that Vitamin E was able to reduce clearly the CL peroxidation (Table 1) and cytochrome *c* release (Figure 7) in 4HPR-treated cells and therefore, these events occurred later than ROS generation. However, we cannot exclude the possibility that other actions of ROS–different from that concerning mitochondria membranes–contribute to caspase activation and therefore to 4HPR-induced cell death.

In summary, our results suggest a linkage between ROS generation at the MRC (between complexes II and III), cardiolipin peroxidation, cytochrome *c* release and caspase-3 activation in 4HPR-induced apoptosis.
